# Beyond the Marrow: Unveiling Uncommon Sites of ALL Relapse with ^18^ F-FDG PET/CT

**DOI:** 10.1055/s-0044-1787894

**Published:** 2024-06-24

**Authors:** Siven Kar, Harshita Gupta, Nusrat Shaikh, Vikram Lele

**Affiliations:** 1 Department of Nuclear Medicine and PET-CT, Jaslok Hospital and Research Centre, Mumbai, Maharashtra, India

**Keywords:** ALL, adult ALL, extramedullary relapse, FDG, MRI

## Abstract

Extramedullary infiltration of acute lymphoblastic leukemia/lymphoma (ALL) to genital organs is extremely rare. Here, we present a case report of an asymptomatic 49-year-old female, known case of precursor B-cell ALL, who was incidentally detected with thickened and heterogeneously hyperechoic endometrium on sonography. Contrast magnetic resonance imaging detected large polypoidal enhancing lesions showing intense diffusion restriction occupying the endometrial cavity and similar lesions in the left adnexa, left ovary, and fallopian tube which were suspicious for leukemic infiltration because of the clinical history and atypical appearance of the lesions. ^18^ F-fluorodeoxyglucose positron emission tomography/computed tomography ( ^18^ F-FDG PET/CT) was done which revealed intensely metabolically active lesion in the endometrial cavity, left adnexa, omental nodules, retroperitoneal lymph node, pancreatic lesion, and few irregular nodules in the right lower lobe. Biopsy findings confirmed extramedullary relapse of ALL. Hence, ^18^ F-FDG PET/CT can act as a good whole body survey to look for extramedullary sites of relapse.

## Key Messages

^18^ F-FDG PET/CT can act as a good modality for a whole body survey to detect bone marrow involvement, detect extramedullary sites of ALL involvement, and also to guide the site for biopsy.

## Introduction

Extramedullary involvement of pelvic and retroperitoneal organs is extremely rare. Female genital organ involvement is usually seen in lymphomas, even then it represents a subgroup of < 1% of all primary extranodal lymphomas. Here, we report a case of acute lymphoblastic leukemia/lymphoma (ALL), with multiple unusual sites of extramedullary relapse detected on an ^18^ F-fluorodeoxyglucose positron emission tomography/computed tomography ( ^18^ F-FDG PET/CT) scan, including the uterus, ovary, fallopian tube, omentum, peritoneal fluid, pancreas, and lungs.

## Case History

**Fig. 1:**
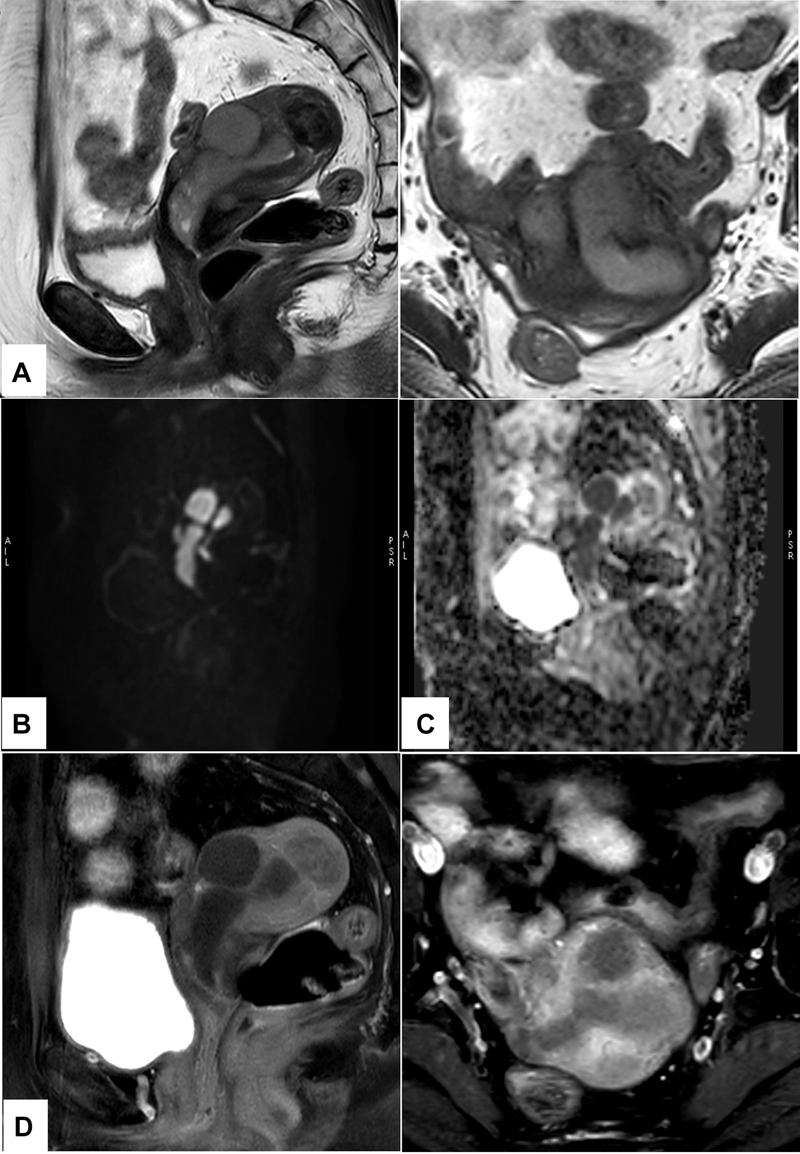
Magnetic resonance imaging (MRI) pelvis showing linear polypoidal lesion within the endometrial cavity. It appears hyperintense on T2-weighted (T2W) sagittal and axial images ( **A** ), intense diffusion restriction ( **B** ) with low apparent diffusion coefficient (ADC) values ( **C** ), and hypointense on T1W sagittal and axial images ( **D** ).

**Fig. 2:**
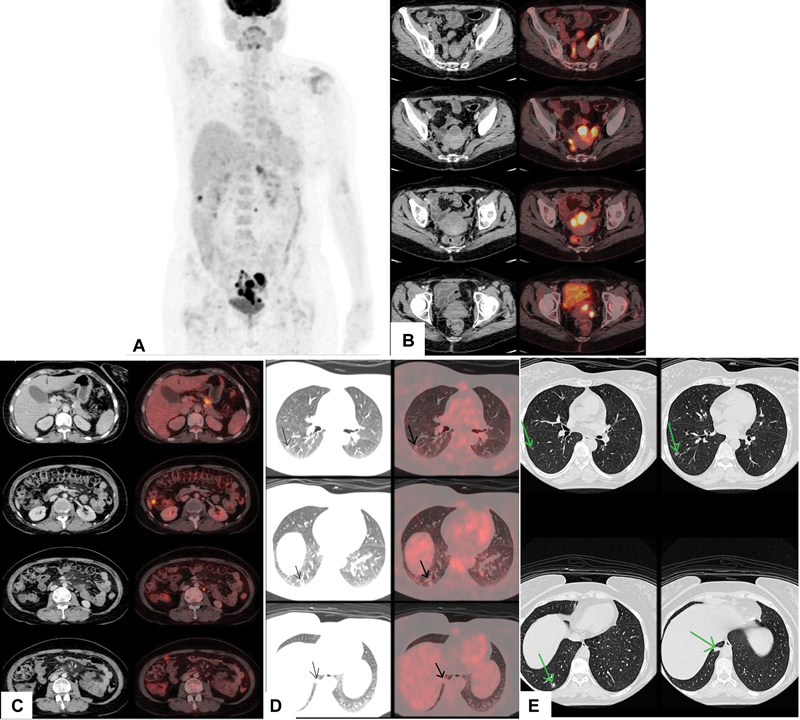
^18^ F-fluorodeoxyglucose positron emission tomography/computed tomography ( ^18^ F-FDG PET/CT) ( **A** ) maximum intensity projection (MIP) image, ( **B** ) axial sections of pelvis showing metabolically active lesions in the uterus, cervix, and left adnexa. ( **C** ) Axial sections of abdomen show metabolically active pancreatic lesion, omental nodules, and retroperitoneal lymph nodes. Axial sections of lung—( **D** ) FDG and ( **E** ) high-resolution computed tomography (HRCT) show irregular ill-defined nodules seen in the superior segment and posterior basal segment of the right lower lobe.

A 49-year-old female, known case of precursor B-cell ALL (postchemo- and radiotherapy), asymptomatic patient, was posted for bone marrow transplant (BMT). Routine sonography for screening before BMT showed thickened and heterogeneously hyperechoic endometrium and endocervix with multiple echogenic air foci within, with evidence of vascularity within the endocervix. Magnetic resonance imaging (MRI) pelvis with contrast was advised to rule out any neoplastic etiology. MRI pelvis revealed large polypoidal mildly enhancing lesion showing intense diffusion restriction occupying the endometrial cavity and extending inferiorly to the cervical canal within the myometrium ( Fig. 1 ). These lesions were of atypical appearance and enhancement, hence a possibility of uterine infiltration by leukemia was considered over endometrial carcinoma. Similar lesions were seen in the left adnexa involving the fallopian tube and ovary. Further imaging with whole body ^18^ F-FDG PET/CT was done. It showed high-grade metabolically active hypoenhancing hypodense lesion within the endometrial cavity extending into the cervical canal with few other hypodense lesions in the uterine wall along with heterogeneously enhancing lesion involving the left adnexa, few omental soft tissue nodules, and subcentimeter-sized para-aortic retroperitoneal lymph node ( Fig. 2 ). Another metabolically active hypodense lesion was found in the body of pancreas and few irregular nodules were seen in the lower lobe of right lung with one of them showing cavitation within, which we reported as likely part of the same disease process. Biopsy correlation was advised. The patient underwent laparoscopy-guided biopsy and specimens were taken from the left fallopian tube, left ovary, endometrial curettage, and peritoneal fluid. Findings were reported as atypical lymphoid cells which were diffusely positive for PAX5, CD79a, and CD10. Many of these were positive for Tdt and CD20. The lymphoid cells were immunonegative for CD3 and MPO. The K _i_ -67 proliferation index was approximately 100%. Hence, a diagnosis of extramedullary relapse of precursor B lymphoblastic leukemia/lymphoma was made.

## Discussion

ALL is the malignant clonal proliferation of early, abnormal, immature B or T lymphocytes. This can eventually replace the normal hematopoietic cells of the bone marrow and other lymphoid organs. The French–American–British has classified it into three types (L1, L2, and L3) depending on the cell morphology. It is the most common cancer in the pediatric age group. It typically affects children in the age group of 2 to 15. Adult ALL is rare with poorer prognosis. It usually affects those above 40 to 50 years of age. It is the second most common leukemia affecting adults.

Patients present clinically with constitutional B symptoms (fever, night sweats, and weight loss), anemia, frequent or easy bruising, frequent infections, dyspnea, etc. Treatment is done in two phases—first is remission induction therapy (to kill the leukemia cells in the blood and bone marrow) and then is postremission therapy (to kill any remaining inactive leukemic cells which may cause future relapse). Newer and more efficacious treatment protocols, use of multiagent chemotherapy, and improved risk stratification of patients have led to good overall survivability of patients. However, many do relapse, especially adults, because of their poorer prognosis (∼50%). 1

Most commonly relapse is seen in the medulla of the bone. The most common extramedullary involvement of ALL include lymphadenopathy, hepatosplenomegaly, central nervous system (CNS), skin, renal, and orbital involvement. 2 Relapses are much more common in CNS and gonads (testes), as they are the “sanctuary sites” because of the relative protection of these sites from chemotherapy drug delivery due to tissue–blood barrier. 3 Although less common than testes, ovaries may also be involved. Occasional case reports have mentioned rarer sites of relapse including the pancreas, breast, and soft tissue/muscle. 4 In our case report, we present many such unusual sites of relapse, including the uterus, ovary, fallopian tube, omentum, peritoneal fluid, pancreas, and lungs. According to literature, the omentum and ovary can attract B cells with more 5T4 oncofetal antigen transcript. 1

^18^ F-FDG PET/CT although not commonly indicated in leukemia, has been used to look for bone marrow involvement (focal or diffuse), guide the site of biopsy aspiration, for prognostication, and to look for extramedullary involvement. 5 CT and MRI have been the traditional modalities to detect such lesions; however, FDG can detect small and occult lesions and also provide a whole body survey which the abovementioned modalities fail to do. It can also be used to predict and monitor response to treatment as metabolic response occurs earlier than morphologic regression in size. 6 It has also been used to detect Richter's transformation of chronic lymphocytic leukemia.
